# Edge cases in animal research law: Constituting the regulatory borderlands of the UK's Animals (Scientific Procedures) Act

**DOI:** 10.1016/j.shpsa.2021.09.012

**Published:** 2021-12

**Authors:** Alexandra Palmer, Reuben Message, Beth Greenhough

**Affiliations:** a School of Geography and the Environment, Oxford University Centre for the Environment, University of Oxford, Oxford, UK; b School of Biological Sciences, The University of Auckland, 3a Symonds Street, Auckland 1142, New Zealand

**Keywords:** Animal research, Borderlands, Boundary work, Law, Regulation, Risk management

## Abstract

This paper explores how the boundaries of the UK's Animals (Scientific Procedures) Act (A(SP)A) are constituted, as illustrative of the rising importance of legal procedures around animal research and how these are continuously being challenged and questioned. Drawing on empirical work in animal research communities, we consider how it is decided whether activities are undertaken for an “experimental or other scientific purpose”. We do this by focusing on “edge cases”, where debates occur about whether to include an activity within A(SP)A's remit. We demonstrate that the boundaries of animal research regulation in the UK are products of past and present decisions, dependencies, and social relationships. Boundaries are therefore not clear-cut and fixed, but rather flexible and changing *borderlands*. We particularly highlight the roles of: historical precedent; the management of risk, workload, and cost; institutional and professional identities; and research design in constituting A(SP)A's edges. In doing so, we demonstrate the importance of paying attention to how, in practice, animal law requires a careful balance between adhering to legal paragraphs and allowing for discretion. This in turn has real-world implications for what and how science is done, who does it, and how animals are used in its service.

## Highlights

• UK animal research law covers activities done for “an experimental or other scientific purpose”.
• Whether this criterion is met is debated at “edge cases”.
• Examples: medicine production, wildlife tracking, fisheries and veterinary clinical research.
• Legal boundaries flexible, shaped by past/present decisions and social relationships.
• Key factors: precedent, risk/workload/cost management, identity, study design.

## 1 Introduction

Historically and today, the law serves as a site of struggle over animal research. Disputes over legal regulations in 19th and 20th century Norway reflected fundamental questions about who should have a say over what and how science is done, and how to ensure that science serves society (Asdal, 2008). Similarly, the UK's Animals (Scientific Procedures) Act (A(SP)A) of 1986 sought to balance the interests and concerns of scientists, vets, animal welfare campaigners, and publics (Hollands, 1995; Myelnikov, 2019). If we accept that the law plays this central role in balancing concerns about animal research, it is important to understand how this is facilitated in practice through the interpretation of key terms within the legislation such as “animal research” and “experimentation”. Our purpose in this paper is to explore how the remit and edges of animal research law are *constituted*, in the sense of grasping something in accordance with cultural codes that make it meaningful for a particular group (Valverde, 2003), in order to determine what counts as permissible animal research and experimentation.

In asking this question, we avoid the big philosophical problems associated with how to define either law or science. While thinkers have long wrestled with the so-called “problem of demarcation” (i.e. how science should be distinguished from non-science), science studies has demonstrated that demarcation, so difficult in theory, “is routinely accomplished in practical, everyday settings” (Gieryn, 1983, p. 781; see H. M. Collins & Evans, 2007; H. M. Collins & Pinch, 1982; Gieryn, 1995 for examples). Following in this deflationary tradition, we are less concerned with the problem of how “science” ought to be defined than with how actors in the past and present have decided what counts as a “scientific procedure” for the purpose of regulating animal research. It is thus law's boundaries more than the boundaries of “science” that are our concern—though our argument suggests that they are co-constitutive (Jasanoff, 2008). We are therefore primarily interested in how the law works in action: how the law's boundaries are shaped by historical decisions that intervene in the present; pressures such as risk and workload mitigation; social relations and identities; forms of knowledge (e.g. administrative expertise) (Valverde, 2003); and the discretion exercised by individuals, such as through “tailoring” (Calvert, 2006).

To do this, we focus on the UK's A(SP)A, which is regulated by a specialised unit within the UK's Home Office: the Animals in Science Regulation Unit (ASRU). To require an A(SP)A licence, an activity must be: (i) performed on a “protected animal”, i.e. living vertebrates and cephalopods beyond a certain stage of development; (ii) be judged to meet the “lower threshold”, defined as causing the animal “a level of pain, suffering, distress or lasting harm equivalent to, or higher than, that caused by inserting a hypodermic needle according to good veterinary practice” (Home Office, 2014, p. 10); and (iii) be undertaken for education or “an experimental or other scientific purpose”. Exploring how this third criterion is interpreted and implemented is the subject of this paper. However, what “experimental or other scientific purpose” means is not explicitly defined, either in A(SP)A itself or its associated guidance. Rather, these documents offer lists of inclusion and exclusion criteria: several activities are identified as outside of A(SP)A's remit (e.g. recognised veterinary practice), and any research conducted under the Act must fall into at least one of the categories listed under “permissible purposes” (e.g. basic, translational, and species conservation research). In short, A(SP)A offers lists of inclusion and exclusion criteria, but little other detail on where to draw the boundaries between what counts as a regulated scientific procedure and what does not. Furthermore, in the process of deciding whether a specific piece of work with animals constitutes a regulated scientific procedure, a whole series of other boundaries are also invoked, including those between research and medicine testing, animal conservation, husbandry, and veterinary treatments, as well as between science and non-science.

It is important to note that the question of whether an animal research project falls within A(SP)A's remit and therefore requires licensing under the Act does not normally arise. Biomedical research using mice, for example, generally requires an A(SP)A licence even if certain parts of the research process (e.g. posthumous tissue collection) are not directly regulated under the Act. However, in some cases researchers do need to ask whether their work requires an A(SP)A licence at all. This question arises in what we term “edge cases”, which most commonly emerge when scientific research closely resembles, relates to, and/or is intermingled with activities that challenge particular, canonical views of “science”. Examples include activities that straddle the border between research and manufacture of medicines, such as the production and testing of medicinal antibodies. Edge cases also arise in settings where there is a fine line between research and activities that are excluded from A(SP)A's remit, such as animal husbandry (e.g. in agricultural, fisheries, and zoo-based research), veterinary treatment (e.g. in veterinary clinical trials), and identification (in the case of tagging wildlife). We draw on all of these examples to our make our case.

Exploring how A(SP)A's boundaries are drawn is an important task because it illustrates how the interpretation and application of legal procedures has real-world implications for animal research and welfare, in terms of what and how science is done, who does it, and how animals are used in its service within the UK. In this way, the UK A(SP)A case speaks to issues around animal research law in other contexts, for example in Sweden where the legal boundary between research and animal husbandry is similarly complex (Lindsjö et al., 2019). We demonstrate that in practice, the boundaries of regulated animal research are products of complex, intertwined past and present decisions, dependencies, and social relationships. Boundaries are therefore not clear-cut and fixed, but rather flexible and changing *borderlands*. Drawing on Hinchliffe et al.’s (2013: 538) reading of Sassen's (2008) work, borderlands are places where “where borders are continually being restated through the juxtaposition of different elements, some close up, others folded in from afar, detached and re-embedded in ways that give rise to new and novel arrangements through different types of engagement”. Thus, while we use terms such as “boundary”, “border”, and “edge” throughout this paper, these terms should not be taken to imply firmness or fixedness. We show that multiple actors are involved in defining and evolving A(SP)A's boundaries, including regulators, researchers, and the publics and interest groups with which they interact. As such, we follow in the vein of recent research on how the practices and regulation of animal research are historically and socially constituted (see for example Davies et al., 2018; Druglitrø & Kirk, 2014; Kirk, 2010).

After describing our methodology, we explore five key factors that emerged from our empirical research as important in constituting A(SP)A's boundaries. First, in section 3 we consider how historical precedents (i.e. past decisions about edge cases) shape boundaries, yet can also be overturned in light of contemporary challenges or perceived weaknesses with historical regulations. We draw in particular on the example of the production and testing of medicinal antibodies to make this case. We then, in sections 4, 5, consider how the efforts of both regulators and researchers to manage risks (to both animals and institutions), workloads, and costs shape A(SP)A's borders, drawing on examples such as the capture and tracking of wildlife. Section 6 explores the role of professional and institutional identities in shaping whether researchers think of themselves as doing “science”, and whether they believe their institution is equipped to secure A(SP)A licences. In section 7 we then consider how research design and ideas about methodological rigour feature in constituting A(SP)A's edges, with particular reference to veterinary clinical research. In concluding we suggest an appreciation of how A(SP)A's boundaries are drawn in practice may also have implications for future revisions of A(SP)A and its scope, and for animal research and animal welfare more generally.

## 2 Methods

This paper is based on qualitative research within animal research communities in the UK, undertaken as part of an interdisciplinary, collaborative research project titled *The Animal Research Nexus* (AnNex; see www.animalresearchnexus.org/). Central goals of AnNex, and of this paper, are to explore how past and present factors have shaped the social contract around animal research in the UK, and to better understand emerging challenges and issues in the regulation and practice of animal research. This paper draws primarily on data and insights derived from one sub-strand of AnNex, which focuses on social, ethical, and regulatory issues arising when A(SP)A steps out of the laboratory and into various kinds of “field”, such as wildlife field sites, veterinary clinics, farms, fisheries, and zoos.

This study adopted a mixed-methods approach, involving in-depth interviews, participant-observation, and documentary analysis, including analysis of historical sources. We undertook semi-structured interviews with 30 individuals, including researchers, vets, animal welfare advocates, and regulators, and an additional 24 lengthy informal conversations with others. The majority (30, i.e. 58%) of conversations were with researchers, while 10 (19%) were with regulators,^1^ and the remainder with others such as vets involved in research support. This research also involved participant-observation during visits to research projects and events such as conferences and training courses. We thematically analysed interview transcripts, field notes, and relevant documents using NVivo qualitative data analysis software. In addition to these research activities, we engaged in various stakeholder-focused events, including holding a workshop on non-laboratory research where some themes discussed in this paper also arose (Palmer, Greenhough, et al., 2020).

Due to the sensitive nature of the topic, we have adopted a policy of pseudonymisation and de-contextualisation to ensure privacy of participants. All interviews were conducted with written consent from participants. This research was granted ethical approval by the Central University Research Ethics Committee of the University of Oxford (Reference Number: SOGE 18A-7).

## 3 Historical precedent

The first feature that we propose shapes A(SP)A's boundaries is historical precedent, by which we mean past decisions about difficult and “edge” cases. Yet, as we demonstrate, decisions made in the past are not immutable. Judgements always need to be made about how precedents relate to new cases, and precedents may be challenged according to changing contexts and views about animal research and its regulation. We discuss the importance of precedent using the example of the production and testing of medicinal antibodies, which provoked discussion about the legal definition of an “experiment” in the 19th century.

Because it is their job to oversee the implementation of A(SP)A, Home Office inspectors (HOIs) are central to deciding which activities involving animals fall within the remit of the legislation. Indeed, it is a common refrain amongst researchers that “it depends on your inspector” whether marginal projects are deemed to require an A(SP)A licence, implying that decisions can feel arbitrary and subjective. Like many laws, A(SP)A is designed to allow inspectors to apply a degree of judgement based on their “administrative knowledge” derived from passed-down knowledge from other inspectors, job-based customary norms, and intuition (Valverde, 2003, p. 53). Yet while inspectors often do have considerable autonomy in decision making – especially if they are experienced in their roles – they do not act alone, and their decisions are fundamentally shaped by precedent. Inspectors also told us that they meet together, usually monthly, to discuss difficult applications and grey areas. The decisions they make are also “written down, they're filed away for future use” (interview with inspector ‘Gail’, 15 May 2019) and stored in a “database of policy and precedence” (interview with former inspector ‘Craig’, 25 June 2019). These precedents become crucial in adjudicating later cases.

The contents of this “database” are off-limits to researchers, and HOIs are not at liberty to discuss how the instances it contains help to adjudicate on individual cases. However, other sources of established precedent are also critical and can be used to illustrate how precedents generally shape and maintain A(SP)A's boundaries. For example, Kirk (2014) demonstrates that a Home Office licence application, submitted by ecologists Chitty and Clarke for an experiment expected to cause animals stress in 1949, set a precedent for expanding animal welfare law to cover activities causing “distress” or “discomfort” in addition to physical “pain”. Here we draw on a similar case described by Tansey (1989): a debate in the late 19th century about the testing and production of medicinal antibodies that established a precedent for what counted as an “experiment” under A(SP)A's predecessor, the Cruelty to Animals Act (CAA). The case involved both *raising* antitoxins, by injecting diphtheria toxin into horses or donkeys to make them immune and then extracting their blood to create antitoxin medicine, and *testing* antitoxins by injecting small rodents with the antitoxin medicine to test efficacy and safety. The CAA specified that any “experiment must be performed with a view to the advancement by new discovery of physiological knowledge or of knowledge which will be useful for saving or prolonging life or alleviating suffering” (3(1)). The production and testing of medicines were borderline in this respect, since they “cross the conventional boundaries between science, regulation, and industrial innovation”, much like “regulatory” or “mandated” science (i.e. science for policy rather than “pure” academic pursuit: Rothstein et al. 1999, p. 245; see also Salter, 1988).

Initially in 1879, and again in 1894, the Law Officers for the Crown of Opinion concluded that the *raising* of antitoxins – i.e. extracting the blood of horses and donkeys that had been made immune to diptheria – did not fall under the CAA because it was “not for ascertaining of a scientific truth” (p.6). In 1895 they also concluded that *testing* antitoxins by injecting small rodents also fell outside of the CAA. However, this opinion was reversed a few months later, at which point the Officers concluded that while raising antitoxins was not an experiment “because it was done to produce a particular, specified substance and not for scientific enlightenment”, testing did count as an experiment under the CAA “as the Act did not specify that it applied only to work for the advancement of knowledge” (p.7).

It would seem that this verdict stuck, and similar decisions were made regarding more modern forms of similar work, such as the production and testing of vaccines using animals. Thus, in 1979–1980 when the Laboratory Animals Protection Bill (a precursor Bill to A(SP)A) was reviewed by a select committee within the House of Lords, the Committee observed that under the CAA.

No new knowledge is gained from the preparation of vaccines by the infecting of an animal with a micro-organism; so this is not regarded as an experiment. But the testing of vaccines on animals, for potency and safety, is an experiment. (House of Lords, 1980a, p. 19).

However, this established precedent was contested by four groups that presented evidence to the Committee. The Royal Society, Research Defence Society, British Veterinary Association, and the Home Office itself all objected to the narrow interpretation of “experiment” within the CAA and argued for its expansion under the new law. All four groups explicitly mentioned the use of animals for culturing viruses and parasites, or producing immune sera for medicine production, as examples of unregulated practices that they believed should be regulated under the new law because these practices can lead to animal suffering. The Home Office's argument for this proposal was that such unregulated activities “may nevertheless involve discomfort to the animal and it is arguable that such activities should be covered by legislation so that the animals are protected by it” (House of Lords, 1980b, pp. 24–25).

Today, “the development, manufacture or testing of the quality, effectiveness and safety of drugs” and other substances – undertaken for several specified purposes such as disease prevention and treatment – is identified as a “permissible purpose” under A(SP)A (Home Office, 2014, p. 43). Thus, the use of animals for production of antibodies, vaccines, and other biological substances is usually interpreted as being included under the Act, although regulation also depends on other factors such as the species used.^2^ Furthermore, the Nuffield Council on Bioethics (2005, p. 222, Box 13.1) notes that the use of the term “procedure” rather than “experiment” in the wording of A(SP)A is intended to capture “the broad range of events that can affect animals” used in science (e.g. breeding), as well as activities undertaken for “other scientific uses”, rather than just experiments. In short, concerns about the CAA's narrow focus on *experiments* may have directly shaped A(SP)A's wording and scope. This meant that while for a time the precedent established in the late 19th century – that producing medicinal antibodies is not regulated, but testing antibodies is regulated – held, this was later challenged and reversed on the grounds that producing antibodies can significantly harm animals. In other words, growing concerns about animal welfare, in the late 1970s and early ‘80s particularly, prompted subtle changes in the scope of animal research law, leading to a broadening out of what could be understood as “science” for legal purposes.

## 4 Risk management

Interestingly, while the ultimate result of the 19th century debates was that regulatory compliance testing of antitoxin drugs did fall under the CAA, not *all* work that we might describe as “regulatory compliance testing” is regulated under A(SP)A. For example, routine batch testing of vaccines for both veterinary and human drugs is covered by A(SP)A (Cooper & Jennings, 2008), but the clinical trials of veterinary medicines required for marketing authorisations are not. Instead, in the UK such work is managed by the Veterinary Medicines Directorate (VMD) via the Animal Test Certificate (ATC). We propose that this inconsistency relates largely to which activities are perceived – by regulators, the general public, or interest groups – to be “risky”, and what kind of resistance is shown by researchers to further regulation. Batch testing of veterinary vaccines has been flagged by the RSPCA as an area requiring greater oversight given inconsistencies in test requirements, opportunities for reducing the number of animals used, and the potential of such tests to cause harms to animals (Cooper & Jennings, 2008). In contrast, veterinary clinical trials have attracted little attention other than from those advocating for reduced regulation. Various vets have expressed concern that the bureaucratic difficulty of conducting veterinary clinical trials in the UK compared with other EU countries will drive veterinary researchers and medicines manufacturers offshore (Braidwood, 2015; Moens et al., 2007; Nanjiani et al., 2015). Furthermore, a policy review that aimed to streamline the regulation of veterinary clinical trials as part of DEFRA's “Red Tape Challenge” (DEFRA, 2014) attracted feedback from only nine stakeholders, who tended to repeat these complaints about excessive bureaucracy (VMD, 2015). This example illustrates how efforts to manage risks posed to animal welfare, both past and present, and the views of interest groups such as vets, researchers, and animal welfare groups, shape the constitution of A(SP)A's borders.

Two main kinds of risk would appear to be relevant to decisions about whether routine compliance testing and other borderline activities are counted under A(SP)A. First, there is risk to the animals themselves, as raised for example by the Home Office in its submission to the House of Lords Select Committee (House of Lords, 1980b, pp. 24–25). Second, there is political or reputational risk to politicians and officials that their policies will be criticised by the general public or by special interest groups. Often, but not always, the second follows from the first; for example, the RSPCA's concern for animal suffering may lead them to shine a spotlight on an issue and thus raise its public profile. Managing both kinds of risk has been identified by HOIs as shaping their practices. For example, HOI Gail referred to both public perceptions and harms to animals in describing which wildlife projects they prioritise visiting and inspecting:

It depends what people are doing as to how high risk it's deemed to be. […] Badgers are a public concern, there's been a lot of controversy about those projects, so they are likely to be inspected more frequently than the general project that goes on in an establishment. [… On] the other end of the scale, there are people who are doing conservation work on species that perhaps are not so high in the public consciousness and so the relative risk for the minister then is lower than it would otherwise be. They're trying to preserve the species, [and] the likelihood of them doing anything wrong, because of that main concept, is lower. [For example] they may only be, […] capturing them to measure them.

Gail references both forms of risk: potential harm to animals, with “low grade” interventions potentially requiring less attention; and “risk for the minister”, specifically the Home Secretary, which is greater in relation to research activities of “public concern”. She also situates this risk-focused approach in a context where inspector time is limited and inspection policies must therefore be allocated wisely (Message & Greenhough, 2019, p. 13).

Such risk-based approaches to regulation are not unique to the Home Office's ASRU. Rather, risk has been widely promoted as a key concept for regulatory reform efforts, widely referred to as programmes of “better regulation”, within the UK (especially since the 1990s) and internationally. While there has clearly been a push towards risk-based regulation within UK civil service generally, uptake of such policies across departments is variable, and how they are implemented depends on specific institutional contexts and histories (Black, 2005, pp. 512–548; Davies, this issue; Demeritt et al., 2015). As Demeritt et al. (2015: 385) have explained, the pressures of austerity have sometimes encouraged ministers “to do less”, while in other cases “the spotlight of public opinion” encourages ministers “to ‘do more’”. Within the Home Office inspectorate, it would seem that there have been moves to “do less” about perceived low-risk activities. This idea was articulated by both Gail and by former inspector Craig, who similarly spoke of efforts to “tailor inspection schedules probably more towards the things that are likely to be higher risk”, and a tendency within the Home Office to talk about risk management “in terms of protecting minister's back”.

In this context, some of our research participants identified a lack of appetite within the Home Office to extend A(SP)A into perceived low-risk areas, such as the capture and marking of wildlife by citizen scientists. Capture of wildlife is not counted as a procedure under A(SP)A, even when it is undertaken for science, although A(SP)A guidance does allow for some oversight of trapping (e.g. that it is undertaken by a competent person: ASRU, 2016). Furthermore, the “ringing, tagging or marking of an animal, or the application of any other humane procedure for the sole purpose of enabling an animal to be identified” is excluded from A(SP)A so long as it “causes only momentary pain or distress and no lasting harm” (c.14, 2.(8)). This “identification threshold” is more lenient than the “lower threshold” typically applied to determine whether an activity is sufficiently invasive to count as a “procedure“ under A(SP)A (ASRU, 2016). Most wildlife capture and marking activities are therefore excluded from A(SP)A's remit.

Another reason for excluding these activities is that including them could inflate statistics on the number of animals used in scientific research. These figures, published annually by the Home Office, receive a large amount of attention from campaign groups and regularly inform negative press (e.g. Bulman, 2017), petitions against animal experimentation (e.g. Cruelty Free International, 2018), and questions put by Members of Parliament to government ministers (e.g. UK Parliament, 2013: column 34WH). Concern about perceived increases in animal use following enlargements of legislative remit goes back a long way, with Tansey (1989: 8–9) describing similar worries within the Home Office in the 19th century. In the case of wildlife research, the potential for this arises because researchers often catch animals but let them go rather than undertake an A(SP)A-regulated procedure on them, for example if they were the wrong species, age, or sex for the study, or if they appeared to be in especially poor health. Because animals let go after trapping only are currently never counted as “research animals” under A(SP)A, regulating trapping under A(SP)A would increase the number of animals counted in annual statistics, potentially contributing to the risk of criticism.

Risk management, particularly risks of bad publicity directed at institutions, also plays a role in how researchers interact with the borders of A(SP)A, and in particular whether they are incentivised to avoid or embrace undertaking work that would require regulation under the Act. For example, Emma – a professional who was involved in overseeing UK zoo research for about 20 years – when asked how many A(SP)A licences she thinks are held by UK zoos – responded:

None, I think! Officially, none, because most zoos would really shy away from it from a PR point of view. They don't want-, you can't have an A(SP)A in secret, you can't have a secret Home Office licence. It would be known – there would be ways of getting that from an organisation, and from a PR point of view most zoos wouldn't risk doing that. (Interview, 1 November 2018)

Emma noted that a key issue is that “[i]f you say ‘animal research’ people think bad stuff”, meaning that “if some anti-zoo lobby – of which there are many, and we have to be very wary all the time – published a list of zoos that have an animal research licence, it'd be very difficult to counter that negative PR”. Similarly, Margulis (2017, pp. 62, 73), writes that “[i]f the public considers ‘research’ and ‘experiment’ to be synonymous, then this can raise a red flag about ‘zoo research’”. Thus, while some research involving invasive procedures might be perceived as generating positive publicity for zoos, such as cloning endangered species (Friese, 2013), most invasive research is perceived as generating negative publicity and fuelling opposition to zoos by animal liberation and rights activists.

However, avoiding A(SP)A-licenced research can create problems for zoos too, since one of the ways zoos have sought to establish legitimacy as conservation institutions has been to emphasise their role in research (Kaufman et al., 2019; Minteer et al., 2018). Indeed, research is a requirement for European zoos according to the EU Zoos Directive (1999/22/EC) (Reid et al., 2008). According to Emma, in practice zoos tend to restrict their research activities to observational (e.g. examining the effects of new enrichments on animal behaviour) and opportunistic work, such as conducting research using blood originally collected for a veterinary purpose. However, in her opinion this is a “frustrating” situation, since there are many minimally invasive activities that zoos would like to undertake but feel they have to avoid, such as systematically collecting blood before and after a change in diet, or assisting researchers with tracking device calibration using zoo animals. In this way A(SP)A is not only regulating research, but also shaping what kinds of research are perceived to be possible in practical terms.

Meanwhile, we heard from others that they have secured A(SP)A licences for somewhat borderline activities that arguably don't require licensing, as part of their institutions' efforts to appear – in the words of one wildlife researcher – “whiter than white” (AP field notes, 29 October 2018). In a sense, such institutions are, like zoos, managing their relationship with A(SP)A based on public image and risk management. While zoos view securing A(SP)A licences as a risk to their public image, well-regarded universities and government institutions may be more concerned about appearing to be, in the words of wildlife researcher Hugh, “exemplars of good practice, and that means not only are you doing it as well as you can but you need to be seen to be doing it perfectly well” (interview, 12 March 2019). In part this is about managing reputational risk, considered increasingly important in managing not only government bodies but also research universities (Huber, 2011; Power et al., 2009), and in particular the management of their ethical review committees (Hedgecoe, 2016). Thus, risk management influences how both regulators and researchers interact with and shape A(SP)A's boundaries.

## 5 Workload and cost management

As we have seen, regulators may be incentivised to avoid drawing perceived low-risk activities (such as the capture and tagging of wildlife) into the remit of A(SP)A, given efforts to focus on activities most likely to attract criticism. An additional reason to avoid bringing such activities under A(SP)A is workload and cost management, for both regulators and researchers. Wildlife researcher Genevieve reflected that although, in her view, trapping an animal and putting a collar on it involves sufficient harms to animal welfare to be regulated under A(SP)A,

they [the Home Office] don't want to do that [including trapping and collaring wildlife under A(SP)A] because then that has such a huge – which I can understand – that then has such a huge implication across the board on local mammal groups and all this kind of thing. (Interview, 25 June 2019).

Awareness of this concern is longstanding. In a supplementary memorandum submitted during the House of Lords Select Committee hearings leading up to A(SP)A, the Department of Education and Science stated: “if bird-ringing were to be included as a licensable procedure, there are at present 2200 ringers, each operating on average at four sites” (House of Lords, 1980b, p. 239). Thus, extending A(SP)A's scope to include wildlife trapping by citizen scientists would bring in a (perhaps unworkably) large number of additional people for the Home Office to regulate, most of whom would likely be doing work classified as low-risk in terms of animal welfare harms and public concern, and who could also themselves come to represent an unwelcome new pressure group dissatisfied with being regulated in this way.

Researchers themselves actually often described a desire to steer clear of A(SP)A licensing if at all possible, due to the large investment of time and resources that this requires. Wildlife researchers regularly complained about the large number of permits, from a wide array of different institutions, required to undertake their research (Palmer, Reynolds, et al., 2021; see Paul & Sikes, 2013 for similar comments about research in the USA). Researchers also frequently remarked on the time required to secure A(SP)A licences. Veterinary researcher Fred expressed a common sentiment when he observed that “however quick it is it still takes a minimum of about a month and can take six months easy, why would you do that if you could avoid it?” (interview, 27 September 2018). Other researchers spoke of the difficulty of having students work under A(SP)A. For example, wildlife researcher Geoff explained that “we normally say six months” to process a licence amendment that would enable a student to do a new piece of research not already covered in the group's licence, such that by the time the amendment is approved, “probably the student's long gone” (interview, 7 September 2018). Cost, meanwhile, can add up, with all personal licence holders required to undertake training at a cost of hundreds of pounds per person, and HOIs potentially requesting upgrades to facilities or equipment involved in research. For example, wildlife researcher Graham described being asked many years ago by his HOI to upgrade security infrastructure at his research site, which cost several thousand pounds. As a result of this and various other concerns, Graham reported steering clear of any research projects requiring A(SP)A licencing for over a decade, as

it's just become too expensive, too time consuming […] I just thought it's not worth it. So I've compromised. But I genuinely think there are some important questions that I'm not addressing because of that. (Interview, 14 September 2018).

While Graham's complete abandonment of all A(SP)A-licenced research is an extreme case, other researchers spoke of avoiding AS(P)A by modifying their project design to make it clearly sub-threshold, or abandoning projects altogether. Participants also occasionally expressed concern that some researchers may avoid A(SP)A via more devious means, such as by exploiting loopholes, though no participants described doing this themselves.

There is therefore an incentive for wildlife researchers to avoid undertaking work that requires an A(SP)A licence if possible. Thus, contrary to Gieryn’s (1983: 781) argument that scientists enjoy “considerable material opportunities and professional advantages” over non-scientists, when it comes to working with animals in the UK, and in other countries such as Sweden (Lindsjö et al., 2016), it may sometimes be easier to not be viewed as a scientist when it comes to being regulated under science-focused animal research law. Indeed, research participants observed that while it is remarkably easy for non-scientists (e.g. farmers and anglers) to harm animals, using animals specifically for science is rather difficult given the time and resources involved in securing A(SP)A licences (see also Hobson-West, 2012).

## 6 Institutional and professional identity

The incentive to avoid A(SP)A licensing may be even stronger for non-university researchers – such as citizen scientists and those based in zoos and teaching-focused agricultural colleges – since their institution (or lack of institution, in the case of citizen scientists) would be unable or unwilling to secure a licence due to cost and a lack of institutional expertise. For example, Calum, who is involved in the capture and tracking of birds, argued that requiring A(SP)A licences for such work “would scupper most of it” due to the difficulty of securing licences for citizen scientists (interview, 1 March 2020). Similarly, in explaining why zoos avoid doing research that would require an A(SP)A licence, Emma noted that

it's just amazingly complicated and bureaucratic, and they [zoos] just don't have the resources to manage it. I've been on ethics committee for [a company], which was a big lab […] and their ethics committee is entirely about reviewing the Home Office licences. The paperwork is … Most zoos do not have the resources to manage that.

Thus, researchers linked their sense of being able to secure A(SP)A licences to their institution's resources and familiarity with the “knowledge format” of the A(SP)A licence application (Valverde, 2003).

Institutional knowledge (or lack thereof) may also have another important effect, namely whether researchers are even aware of A(SP)A's existence and scope. Many participants reflected that potentially only certain groups are aware of the law and therefore think to “ask the question” about whether they need an A(SP)A licence. As summarised by wildlife researcher Genevieve, “I think if you worked in a group which didn't have licences you probably wouldn't ask the question”. The Home Office is clearly aware of this risk. For example, former HOI Heather explained that there is a fine line between fisheries research and *management*, the latter being excluded from A(SP)A. Heather described a “rule of thumb” for distinguishing research and management as “it's not about what you do, it's about the fact that you have a plan”: “so if you have a plan about what you're doing depending on the results, it's probably fisheries [management] because you've got an action plan and you know what you're going to do.” For example, if you are validating a model about how to manage river systems, a pertinent question is: “are you managing similar river systems from your information?” If the answer is genuinely *yes* then “that is still management”, whereas “[i]f you haven”t built that model yet, if you are validating that model, it's science”. This distinction means that if a model becomes sufficiently validated that it is used as a foundation for management plans it can move from being A(SP)A-regulated research to fisheries management. However, Heather acknowledged that fisheries managers might not be aware of this distinction and therefore might not realise that they are doing science, noting that “I would have guessed that two thirds of fisheries trusts didn't even know the Home Office [ASRU] existed”. However, she pointed out that this is “not a problem with A(SP)A” per se, but rather of ignorance among fisheries managers, potentially at an institutional level. While arguably fisheries managers (or, at least, their institutions) have a “duty to know” about A(SP)A and its definition of research (Valverde, 2003), in practice little can be done to enforce this duty: inspectors indicated that they always follow up when they hear about someone who should be regulated but lacks a licence, but realistically they only know about the activities of “the people who talked to us” (interview with ‘Heather’, 17 January 2019).

The importance of institutional knowledge is demonstrated by the key regulatory role played by local Animal Welfare Ethical Review Bodies (AWERBs): legally mandated committees with a number of specified tasks and membership requirements. AWERBs are widely acknowledged to be crucial in deciding what research is licensed, because they are tasked with advising establishment licence holders whether to support or reject project proposals before they are sent on to the Home Office (Home Office, 2014). AWERBs can thus act as gatekeepers with respect to both the content of applications destined for the Home Office, and what reaches the desk of a HOI in the first place. Some UK institutions also run avowedly non-A(SP)A research with animals past their AWERBs, or in some other manner combine their legally mandated AWERB business with a local policy of broader ethical review. In other cases, institutions that undertake research with animals but pointedly avoid anything requiring A(SP)A licencing may still have ethical review bodies of their own making. Such non-A(SP)A review bodies, or AWERBs that also consider non-A(SP)A work, may therefore be in a position of deciding whether to enquire with their local HOI about whether a project requires an A(SP)A licence. However, if an institution lacks an ethical review body, or its ethical review body has little knowledge of A(SP)A, then researchers at the institution may receive little assistance not only in crafting A(SP)A licence applications, but also in determining whether their work falls within A(SP)A's remit. Citizen scientists are in an even more difficult position given that they by definition lack institutional affiliation, making processes such as ethical and regulatory oversight challenging (Palmer, Reynolds, et al., 2021). As Valverde (2003) observes, the licence as a governance technology primarily acts as a way of minimising opportunities for violations of the law, rather than directly punishing people for crimes. The goal is to restrict when, where, how, and by whom certain activities can be undertaken. In the case of A(SP)A, the tool of licensing may (unintentionally and indirectly) exclude certain kinds of researchers such as citizen scientists from undertaking regulated animal research, given the institutional knowledge required to secure a licence.

In addition to institutional knowledge, the related matter of professional identity was often highlighted as leading to inadvertent self-exclusions from A(SP)A. For example, former inspector Colin – himself a vet by training – proposed that vets may struggle to distinguish recognised veterinary practice (RVP), which is excluded from A(SP)A, and research (*emphasis added*):

vets get confused at the interface between veterinary clinical practice and veterinary research, and they say, “Well we're just taking a blood sample, I do that every day of the week.” I'm saying, “Ah *you're doing it for a different purpose now*, you're not benefiting the animals by taking this sample therefore you need to apply all these other rules,” and they just kind of roll their eyes at you and say, “Hmm.” (Interview, 26 June 2019)

As Colin argued, this is why it's important to have some lectures in vet school on A(SP)A, “so that when they come out, if nothing else they ask the question”. Colin here speaks of the risk that professional identity might shape how people think about whether their work counts as “science”. Similarly, zoo researcher Emma suggested that “my general impression of vets is that they feel they're allowed to do stuff because they're vets”. In other words, there is a worry that vets assume they are doing RVP, or otherwise working under the Veterinary Surgeons Act (1966), simply because of their profession. Meanwhile, former HOI Heather suggested that you might have “interested marine biologists who have gone to work for a fisheries trust” who also don't think to ask about regulation, because they have come to think of themselves as involved in fisheries management.

In other words, participants sometimes suggested that there is a tendency to conflate *purpose* and *profession,* while A(SP)A is intended to consider only the former. This slippage was conveyed by university-based fish researcher Evan, who referenced his identity as a scientist and the activities he was undertaking to some extent interchangeably:

So my interpretation, which might be wrong, is that if the procedure's happening anyway, so if a fisheries manager or shepherd or whatever, is dealing with animals and doing something to them and you're simply standing there collecting the data, then that wouldn't be A(SP)A. But if the only reason it's happening is because you're doing some experiment or you as a scientist … Especially, I've been told that, you know, well you're from a university therefore it must be science. You're not doing it for husbandry reasons. (Interview, 15 January 2019)

Evan indicates that while he is well aware of A(SP)A's focus on scientific *purpose*, profession also indirectly comes into assessments about licensing: given that he works for a university, how could anything he does *not* be science? Thus, the shared identity of scientists – marked by things like formalised training, shared publication in specialised journals, and location in specific institutions – and similar identities developed in other professions such as among vets, could potentially result in people involved in edge cases including or excluding themselves from A(SP)A.

## 7 Research design

While earlier sections identified reasons why researchers might want to avoid their work being under A(SP)A, in other cases an A(SP)A licence was seen to confer advantage. We saw this, for example, in cases where highly-regarded research universities and government institutions secured A(SP)A licences for projects that arguably didn't require this as a way of managing reputational risk. In addition, some researchers spoke of A(SP)A licences as a mark of credibility and reliability. For example, veterinary clinical researcher Charles observed that not only does going through A(SP)A licensing help ensure that he feels “confident that we're […] doing the right thing by people and the patients”, but it also provides a mark of credibility for drug companies seeking to undertake clinical trials. Comparing the regulatory ease of conducting veterinary clinical trials in the US and the UK, Charles reflected that “it is easier to do these things in the States but do you know, I think pound for pound you get more bang for your buck in the UK” (interview, 27 June 2019).

Thus, working under A(SP)A can confer an impression of work as methodologically rigorous. This impression may derive from the fact that A(SP)A is in many ways concerned with scientific method and research design. The harm-benefit analysis, which is central to A(SP)A's process, requires assessing benefits of research. This assessment in turn involves screening for research integrity and good experimental design (ASC, 2017). In other words, A(SP)A licences are not meant to be granted for all science involving the use of animals, but only for *good science,* which is considered sufficiently likely to reveal important information that harms to animals are considered justified. Thus, insufficiently rigorous studies should (in principle) fail the harm-benefit analysis, and on these grounds be declined A(SP)A licences. Consequently, despite the focus on “scientific purpose” embedded in A(SP)A, ideas about methodological rigour are also a part of how the borders of A(SP)A are established.

HOIs might even decide that methods are so poor that the work cannot count as “science”. This idea very often came up with reference to experimental or highly novel veterinary treatments, which sit in a grey area between science and RVP because they are ideally intended to simultaneously advance the science of veterinary medicine (and perhaps human medicine), and have therapeutic benefit for the animal patient/research subject (Palmer, Skidmore, & Anderson, In Review). The problem, in the opinion of veterinary professional Grace, is that such work is often “experimental but it's not an experiment” (interview, 28 January 2019). Veterinary professional Elaine elaborated on what she perceives to be the issue:

So for instance, if you have a single case – so a dog that is sick – and you want to perform a procedure on it that has never been done in a dog before, that has been done in a human before, and you may have good reason to believe that it would be successful in that dog, that's an experimental procedure. It is not recognised veterinary practice, because it's never been done in a dog before. So it can't be done under RVP, but it can't be done under A(SP)A [either] because it's not for a scientific purpose. (Interview, 11 January 2019)

Indeed, former HOI Heather confirmed that from the Home Office's perspective such work does not count as scientific research, due to the lack of rigour and scientific outputs:

So some of these things are not able to be regulated under A(SP)A because they're not actually science, because actually if you put them forward as an inspector I would have bumped it and said, “Your science is too poor, I'm not going to let you do it.”

Thus, according to Heather such research might potentially be allowed to proceed under the Veterinary Surgeons Act, but it “couldn't go in the research bucket because this is not proper research”. Thus, experimental design (or the lack thereof) is considered in deciding which projects fall within A(SP)A's remit.

The relationship can potentially also go in the opposite direction. An example of this came up during an extended informal conversation with a veterinary clinical researcher who was performing a novel treatment on companion animals (AP field notes, 18 Jan 2019). As he articulated, his work falls into a grey area because it is intended for the animal's benefit, but it is also novel, and he is hoping to build material for publication based on a series of cases. While his institution's ethics board proposed that it should be okay to conduct the project under RVP, the Royal College of Veterinary Surgeons (RCVS), which is responsible for defining RVP, indicated that he should approach the Home Office as it would probably fall under A(SP)A. After requesting to view the researcher's evidence for thinking the treatment would work, the Home Office countered by arguing that there was sufficient support to believe this would benefit individual animals, meaning the project should fall under RVP. Eventually, the researcher was permitted to proceed with his work under RVP. He reflected that while he is certainly happy that the situation was resolved and he was able to proceed, he would have liked to make the project more “scientific” by increasing the sample size through more active recruitment and soliciting peer review, but has been to some extent prevented from doing so by the project's classification as veterinary treatment rather than science. Thus, research design can play a role in shaping A(SP)A's borders with RVP, in that rigorous methods are required for securing an A(SP)A licence, and in turn work classified as RVP assumes – and perhaps demands – less rigorous methods.

## 8 Conclusion

As we have argued, the boundaries of UK animal research law are shaped by historical precedent; risk, workload, and cost management; institutional and professional identity; and research design and perceptions of methodological rigour. Historical precedent, as we demonstrated in section 3, plays an important role in constituting A(SP)A's edges; however, precedents may be challenged in light of changing perceptions of animal research and its regulation, including perceived risks to animal welfare. Risk management also plays a key role, with regulators encouraged to oversee certain practices that are perceived as risky, hence the inclusion of antibody production and testing under A(SP)A, and the broadening of A(SP)A's scope beyond just “experiments” compared with its predecessor the CAA. On the other hand, there is little incentive for regulators to include the capture and tracking of wildlife under A(SP)A, since such work is perceived as low-risk in terms of animal welfare and public attention, and including it could both inflate animal use statistics (potentially drawing negative public attention) and overload already busy inspectors.

Meanwhile, researchers may be concerned about negative public attention from either securing A(SP)A licences (as in the case of zoos) or from *not* securing A(SP)A licences for “edge cases”, as with highly regarded universities and government research institutions. They may also be concerned about workload and cost, with the process of securing A(SP)A licences regularly described as expensive, challenging, and time-consuming. Researchers may also believe that their institution lacks the experience and knowledge required to successfully apply for A(SP)A licences. In some cases, researchers and their institutions may even be unaware of the existence and remit of A(SP)A, potentially leading to unintentional self-exclusions. Despite these disincentives to conduct work under A(SP)A, researchers in other cases saw an A(SP)A licence as a sign of methodological rigour and high standards. On the other hand, the potential to undertake experimental work might suffer if researchers' work is classified by regulators as non-scientific, illustrating that research design and perceptions of methodological rigour play a role in shaping A(SP)A's borders.

Thus, the borders of A(SP)A – and particularly the meaning of “experimental or other scientific purpose”, which has been our focus in this paper – are constituted flexibly by past and present actors, who are incentivised to include and exclude certain practices from the scope of animal research law. We do not point out this flexibility simply to identify a contradiction in the application of A(SP)A; that A(SP)A can be flexibly applied to meet on-the-ground challenges is one of its great strengths. It is therefore more useful to think of the boundary between regulated and non-regulated work under A(SP)A as less of a firm line that can be easily (or even uneasily) drawn, but more as a regulatory borderland. Within this borderland, researchers, regulators, and others involved in animal work can adapt – or “tailor” (Calvert, 2006) – their work towards or away from more regulated scientific procedures. They may do this in response to the specific juxtaposition of precedent, professional (or other) identity, purpose, risk, workload, and so on. However, it is also true that in some cases people help to shape A(SP)A's boundaries unconsciously; actors may be unaware of A(SP)A, or following established scripts based on assumptions about their professional identity or historical precedent.

We suggest that the constitution of A(SP)A's edges, and in particular how “science” is understood and used to define the Act's remit, demonstrates the importance of paying attention to the ways in which legal procedures shape what and how science is done, who does it, and how animals are used in its service. As we have seen, researchers say they often avoid doing certain kinds of studies that they believe would be important or interesting because of a perceived difficulty in getting A(SP)A licencing. Wildlife researcher Graham, for example, explicitly stated that “there are some important questions that I'm not addressing” due to his reluctance to work under A(SP)A. It could be debated whether this is a positive or a negative situation. Some might lament that important research ends up being done offshore due to the difficulty of conducting it in the UK (as we saw in the case of veterinary clinical trials: section 4). Indeed, there is arguably a need for future research examining how differences in international legislation shape the broader global distribution of animal research. On the other hand, you could see the incentive to avoid A(SP)A as positive in that researchers are clearly being encouraged to minimise harm to animals and animal use, provided they do not try to do the work under non-A(SP)A auspices. Furthermore, it should be noted that not all research with animals is discouraged. Rather, regulators might be under pressure to “do less” about overseeing perceived low-risk projects.

Pragmatic decisions about what not to regulate are important, shaping not only what research gets done, but also who does it. Certain institutions and individuals may face greater barriers to entry than others. For example, students and researchers based in institutions such as zoos and teaching-focused agricultural colleges and citizen scientists are among those who would likely struggle to secure A(SP)A licences, due to limitations in both resources and expertise (see Palmer et al., 2020, Palmer et al., 2021). Furthermore, the flexible constitution of A(SP)A's edges can shape *how* science is done, with scientific rigour (or at least replication with a statistically significant number of subjects) not only encouraged for projects falling under A(SP)A, but potentially also discouraged if it is desired that a project classified as non-science remains so. We therefore propose that understanding how the boundaries of animal research law are constituted is not merely of academic interest, but also has important real-world implications for animal welfare and animal research.

## Funding

We gratefully acknowledge the 10.13039/100010269Wellcome Trust for funding this research (Collaborative Award WT205393/A/16/Z).

## Declaration of competing interest

None.

## Footnotes

1 As we show in section 5, Home Office inspectors, who are responsible for overseeing A(SP)A's implementation, are stretched for time and resources. We therefore only spoke with one current HOI, along with three former HOIs. Other regulators were involved with overseeing other laws affecting wildlife and veterinary clinical research.

2 For example, the blood of Atlantic horseshoe crabs (*Limulus polyphemus*) is regularly used for testing vaccines and other pharmaceuticals for endotoxin contamination (Gorman, 2020). However, invertebrates other than cephalopods are not counted as “protected animals” under A(SP)A, and thus bleeding horseshoe crabs for safety testing is not counted as a procedure under the Act.

## References

[bib1] Animals in Science Committee (2017).

[bib2] Asdal K. (2008). Subjected to parliament: The laboratory of experimental medicine and the animal body. Social Studies of Science.

[bib3] ASRU (2016).

[bib4] Black J. (2005).

[bib5] Braidwood J. (2015). Approach to issuing animal test certificates for veterinary medicines. The Veterinary Record.

[bib6] Bulman M. (2017, July 13).

[bib7] Calvert J. (2006). What's special about basic research?. Science, Technology & Human Values.

[bib8] Collins H.M., Evans R. (2007).

[bib9] Collins H.M., Pinch T.J. (1982).

[bib10] Cooper J., Jennings M. (2008).

[bib11] Cruelty Free International (2018). https://petition.parliament.uk/petitions/219512.

[bib12] Davies G., Greenhough B., Hobson-West P., Kirk R.G.W. (2018). Science, culture, and care in laboratory animal research: Interdisciplinary perspectives on the history and future of the 3Rs. Science, Technology & Human Values.

[bib13] DEFRA (2014).

[bib14] Demeritt D., Rothstein H., Beaussier A.-L., Howard M. (2015). Mobilizing risk: Explaining policy transfer in food and occupational safety regulation in the UK. Environment and Planning A: Economy and Space.

[bib15] Druglitrø T., Kirk R.G.W. (2014). Building transnational bodies: Norway and the international development of laboratory animal science, ca 1956–1980. Science in Context.

[bib16] Friese C. (2013).

[bib17] Gieryn T.F. (1983). Boundary-work and the demarcation of science from non-science: Strains and interests in professional ideologies of scientists. American Sociological Review.

[bib18] Gieryn T.F., Tauber A.I. (1995). Science and the quest for reality.

[bib19] Gorman R. (2020). Atlantic horseshoe crabs and endotoxin testing: Perspectives on alternatives, sustainable methods, & the 3Rs (replacement, reduction and refinement). Frontiers in Marine Science.

[bib20] Hedgecoe A. (2016). Reputational risk, academic freedom and research ethics review. Sociology.

[bib21] Hinchliffe S., Allen J., Lavau S., Bingham N., Carter S. (2013, October 1). Biosecurity and the topologies of infected life: From borderlines to borderlands. Transactions of the Institute of British Geographers.

[bib22] Hobson-West P. (2012). Ethical boundary-work in the animal research laboratory. Sociology.

[bib23] Hollands C. (1995). Achieving the achievable: A review of animals in politics. Alternatives to Laboratory Animals.

[bib24] Home Office (2014).

[bib25] House of Lords (1980).

[bib26] House of Lords (1980).

[bib27] Huber M. (2011).

[bib28] Jasanoff S., Hackett E.J., Amsterdamska O., Lynch M., Wajcman J. (2008). The handbook of science and technology studies.

[bib29] Kaufman A.B., Bashaw M.J., Maple T.L. (2019).

[bib30] Kirk R.G.W. (2010). A brave new animal for a brave new world: The British Laboratory Animals Bureau and the constitution of international standards of laboratory animal production and use, circa 1947–1968. Isis.

[bib31] Kirk R.G.W., Cantor D., Ramsden E. (2014). Stress, shock, and adaptation in the twentieth century.

[bib32] Lindsjö J., Cvek K., Spangenberg E.M.F., Olsson J.N.G., Stéen M. (2019). The dividing line between wildlife research and management—implications for animal welfare. Frontiers in Veterinary Science.

[bib33] Lindsjö J., Fahlman Å., Törnqvist E. (2016). Animal welfare from mouse to moose—implementing the principles of the 3Rs in wildlife research. Journal of Wildlife Diseases.

[bib34] Margulis S., Donahue J. (2017). Increasing legal rights for zoo animals: Justice on the ark.

[bib35] Message R., Greenhough B. (2019). “But it's just a fish”: Understanding the challenges of applying the 3Rs in laboratory aquariums in the UK. Animals.

[bib36] Minteer B.A., Maienschein J., Collins J.P. (2018). The ark and beyond: The evolution of zoo and aquarium conservation.

[bib37] Moens Y., Brodbelt D., Seymour C., Brearley J., Clutton E., Taylor P. (2007). Use of the medicines cascade. The Veterinary Record.

[bib38] Myelnikov D. (2019).

[bib39] Nanjiani I., Hume D., Fitzpatrick J., Murray J. (2015). “Recognised veterinary practice” in the context of clinical field trials. The Veterinary Record.

[bib40] Nuffield Council on Bioethics (2005). The ethics of research involving animals.

[bib41] Palmer A., Greenhough B., Hobson-West P., Message R., Aegerter J.N., Belshaw Z., Dennison N., Dickey R., Lane J., Lorimer J., Millar K.M., Newman C., Pullen K., Reynolds S.J., Wells D.J., Witt M.J., Wolfensohn S. (2020). Animal research beyond the laboratory: Report from a workshop on places other than licensed establishments (POLEs) in the UK. Animals.

[bib42] Palmer A., Reynolds S.J., Lane J., Dickey R., Greenhough B. (2021). Getting to grips with wildlife research by citizen scientists: What role for regulation?. People and Nature.

[bib43] Palmer, A., Skidmore, T., & Anderson, A. (In Review). When research animals become pets and pets become research animals: Care and death in animal borderlands. 10.1080/14649365.2022.2073465 PMC10540782 38656701

[bib44] Paul E., Sikes R.S. (2013). Wildlife researchers running the permit maze. ILAR Journal.

[bib45] Power M., Scheytt T., Soin K., Sahlin K. (2009). Reputational risk as a logic of organizing in late modernity. Organization Studies.

[bib46] Reid G.M., Macdonald A.A., Fidgett A.L., Hiddinga B., Leus K. (2008). Developing the research potential of zoos and aquaria: The EAZA research strategy.

[bib47] Rothstein H., Irwin A., Yearley S., McCarthy E. (1999). Regulatory science, Europeanization, and the control of agrochemicals. Science, Technology & Human Values.

[bib48] Salter L. (1988). Mandated science. Mandated Science.

[bib49] Sassen S. (2008).

[bib50] Tansey E.M. (1989). The Wellcome physiological research laboratories 1894–1904: The home office, pharmaceutical firms, and animal experiments. Medical History.

[bib51] UK Parliament (2013).

[bib52] Valverde M. (2003).

[bib53] VMD (2015).

